# Amariah Brigham, M. D.

**Published:** 1849-12

**Authors:** 


*Amariah Brigham, M. D.*—Dr. Amariah Brigham was born in the town of New Marlborough, Berkshire county, Massachusetts, on the 26th day of December, 1798, where his father, John Brigham, was also born. His grandfather, Francis Brigham, one of the first settlers of the place, was from Marlborough in Worcester county, a descendant of Thomas Brigham, who came over from England, and settled in Cambridge in 1640. In 1805, the father of Amariah moved to Chatham, Columbia county, N. Y., where he had purchased a farm, and died there in 1809. On the death of his father, the subject of this memoir, who was now eleven years of age, went to reside with an uncle, Dr. Origin Brigham, a highly respected physician in Schoharie, N. Y. Here he hoped long to reside, and to follow the profession of his uncle, for which he had already imbibed a fondness. But it was so ordered in Providence, that in the course of a few years, this beloved relative was removed by death, and the nephew left with limited resources, to seek some new home and employment.

After remaining a short period with his mother in Chatham, having little taste for the farm, and an ardent desire for books and knowledge, he started off alone, at the age of fourteen, for Albany, in pursuit of a livelihood. He soon found a place there, in a book and stationery establishment, where he resided in the family of the proprietor, and found himself happy. He had here abundant access to books, was in the neighborhood of the courts, the Legislature, and public men, and embraced with eagerness every possible means of acquiring knowledge. One who furnishes the material for this part of the memoir, well remembers the enthusiasm with which he would describe men and scenes of the Capitol, on his occasional visits to his mother at Chatham. Though but fifteen years of age, he could describe the person and qualities of almost every man of note who came to Albany, had his own opinion formed on nearly all matters of public interest, and could cite book and chapter for the ground of his opinion.

He often mentioned one little occurrence in connection with the late Daniel D. Thompkins, who was then governor of the state. He was directed, soon after entering on his new employment, to carry some article of stationery to the Chief Magistrate, who resided in a mansion with spacious grounds in front, near the Capitol. After delivering his parcel, and coming down one of the winding paths to the gate, he picked up a new silk handkerchief which had been accidentally dropped. Presuming it to belong to some of the governor's family, he went back and inquired for an owner. The governor soon appeared in person, gave him many thanks for the return of the article, inquired of him his history, and then dismissed him with a cordial shake of the hand, and a generous piece of money. That occurrence he has often mentioned in later years, impressed deeply on his mind two things; the value of strict integrity in boys, and of kind attention towards them by men of prominence. He said he could not be bribed after that to do a dishonest act for all the wealth of the Capitol.

During a three years' residence at Albany, while he had given perfect satisfaction to his employer, he had retained his desire for professional life, and had devoted all his leisure time to reading and inquiry relating to the same. His mother now moving back to his native place in Berkshire, Massachusetts, he soon got released from his engagements, and resided with her, and entered on the study of medicine with Dr. Edmund C. Peet, a distinguished physician, brother of H. P. Peet, Esq., President of the New York Deaf and Dumb Asylum.

Here he resided and studied more than four years, subtracting his winter term, when he taught school; and one year spent in New York, attending lectures. His study, too, was close and thorough, often amounting to twelve hours a day, besides miscellaneous reading.

While he had at this time, when his professional studies commenced, acquired an extensive acquaintance with books, had practised much in composition, and wrote well, he had never, in form, studied English grammar. One who was the teacher of a select school in the place, informs us that he was waited on by the young medical student, with a proposition to be taught the grammar, and wished to have it all done in a single day. A day was given him, and a hard days' work it was, for hundreds of questions had to be thoroughly answered, and different parts of the text book explained. In the evening, several young persons who had spent months in the same study, undertook to examine the pupil of a day, and found, to their surprise, that he had not only reached their position in the study, but had gone beyond them, and could propose and solve difficulties in the language quite too hard for them. Within a few weeks he commenced the teaching of a school, for the winter, in which he had a large class in grammar, and which was so taught, that at the closing examination, both teacher and pupils received high commendation.

In prosecuting his medical studies, he found that many things which he wanted were locked up in the French language. With the same resolution which had led him to master the English grammar, he procured dictionaries and other helps, and without any teacher mastered the French. Nearly one-third of his large library left, is in this tongue, and was read, in later years particularly, with as much facility as his own vernacular.

The year 1820, when his professional studies closed, he spent with Dr. Plumb, of Canaan, Conn., engaged, most of the time, in practice with him. In 1821 he commenced practice by himself in the town of Enfield, Mass.

Here he remained for two years with fair prospects, but finding a more inviting field before him in Greenfield, the shire town of Franklin county, he removed thither, and practised for two years, when he went to Europe. After a year's residence in France, Italy, England and Scotland, he returned to Greenfield, but moved in April, 1831, to Hartford, Connecticut. Here he had a large and sucessful practice, much of it in the line of surgery, until 1837, when he moved to New York and lectured one winter in the Crosby street Medical College. But his health here not being good, and not liking the confinement, to which he was so unused, he returned, in October, 1838, to Hartford, a place which was always dear to him, and where he had hoped, even the last year, to spend the evening of his days. Dr. Brigham was married Jan. 23, 1833, to Susan C. Root, daughter of Spencer Root, Esq., of Greenfield Massachusetts, by whom he had four children, of whom three, with their mother, survive to mourn his death. In Jan. 1840, he was appointed, in connection with Dr. Sumner, to take charge of the Retreat for the Insane, at Hartford, and in July, 1840, was appointed Superintendent of the same.*

In the summer of 1842, Dr. Brigham was appointed Superintendent of the New York State Lunatic Asylum, at Utica. The institution was opened the 16th of January, 1843. From this time until the period of his death, he was unceasing in his devotion to the great cause of humanity in which he was engaged. It is well known that the building first erected, was intended only as a part of the entire establishment, and consequently was not susceptible of such an arrangement as was necessary for a proper classification. It was the ambition of Dr. Brigham, that the State of New York should have a model institution, and this was impossible without further accommodations; and although his duties were thereby rendered more arduous and responsible, without any increase of remuneration, he was unceasing in his application to the Managers and the Legislature, for additional buildings. In May, 1844, an additional appropriation of $60, 000 was made by the Legislature, to enable the Managers to erect two additional wings for patients, thus doubling the accommodations, and also the necessary room for bakery, wash-rooms, &c., in the rear of the buildings, and thus removing them from the basement of the main building. The new erections were completed in 1846, and were soon filled with patients; from that time until the present, the average number of patients has been from four hundred and fifty to five hundred. Dr. Brigham was not only ambitious of establishing an institution which should be creditable to the state, but, in order that our citizens should avail themselves of its advantages, he labored to diffuse a more extended knowledge of the subject of insanity; this he did by popular lectures, and by embodying in his reports, details of the causes, the early symptoms, and means of prevention, but particularly by the establishment of a quarterly journal, viz., 'The Journal of Insanity,' which was devoted exclusively to this subject. In order to secure its more extensive circulation, it was placed at the low price of one dollar a year, in addition to many copies gratuitously distributed. To the readers of the Journal, nothing need be said of its merits. At the time it was commenced, it was the only Journal of the kind pub-

* The above sketch of the early history of Dr. B. was furnished by a brother of the deceased.

lished, either in this or any other country, and elicited the highest encomiums from the medical and Legal professions, both in Europe and America. Although Dr. B. was the responsible editor, it was the medium of communication for some of the ablest writers in our country. We have reason to know, that in addition to the gratuitous labor of editing and superintending its publication, it was long maintained at a heavy pecuniary sacrifice. In the prospectus to the first number the Doctor says:—

"The object of this Journal is to popularize the study of insanity,—to acquaint the general reader with the nature and varieties of this disease, methods of prevention and cure. We also hope to make it interesting to members of the medical and legal profession, and to all those engaged in the study of the phenomena of mind.

"Mental philosophy, or metaphysics, is but a portion of the physiology of the brain: and the small amount of good accomplished by psychological writers, may perhaps be attributed to the neglect of studying the mind, in connection with that material medium which influences, by its varying states of health and disease, all mental operations.

"We regard the human brain as the *chef d' œuvre*, or master-piece of creation. There is nothing that should be so carefully guarded through all the periods of life. Upon its proper development, exercise and cultivation, depend the happiness and higher interests of man. Insanity is but a disease of this organ, and when so regarded, it will often be prevented, and generally cured by the early adoption of proper methods of treatment."

In August, 1848, Dr. Brigham lost his only son, John Spencer Brigham, a promising and particularly attractive lad of the age of 12 years. In this son was treasured a father's fondest hopes and proudest aspirations. He fell a victim to the dysentery which was prevailing in the Asylum, as also in the neighboring city of Utica, and surrounding country, in a malignant form. A few weeks after, he was called to follow to the grave his only remaining parent. These repeated afflictions, which were felt as parents who have lost the child of their affections, alone can feel, evidently preyed upon a constitution naturally feeble, and seemed to prepare the way for his own premature removal. Though educated by a pious mother, and enjoying the advantages of an early religious education, he, like too many others, had been too much engrossed with the cares of this life to attend much to the future. This circumstance, with some severe strictures in his writings, on the pernicious effects of revivals and protracted meetings on the health of young persons, very unjustly gave rise to a charge of scepticism and infidelity. If there was a fault, it was one into which a medical man like Dr. B., possessed of a strong feeling of benevolence, would naturally run; viz., in his solicitude for the health and physical well-being—to forget that there were other and higher claims than those of this world. For the last four or five years more attention was paid to the subject of religion. The death of his son and mother made him feel more strongly the vanity and uncertainty of all earthly ties, and induced him to place his treasures in heaven. Dr. B. seemed to have a presentiment that his earthly pilgrimage was approaching its termination, and in his letter to his brother, the Rev. John C. Brigham, on the subject of the death of his son and mother, he spoke freely of his own death as not far distant; expressing, however, neither fear or regret. It was but too evident to the friends of Dr. B., that his afflictions, together with his arduous duties, were preying upon a constitution naturally feeble, and he was urged to relax his exertions, and if that could not be done, to resign his situation: but he could not consent to leave his work unfinished, and only promised that when the institution was in a condition to dispense with his services, he would retire, but, alas! that period never arrived. In the month of August, the dysentery again made its appearance in the institution, but in a much milder form than in the preceding year. Dr. B. was seized with diarrhœa, which in many cases was the precursor of the more formidable affection. He however still persisted in discharging the duties of his office, and attending to his patients, until so far exhausted that it was impossible. The writer first saw him on the 27th of August; he had then been confined to his bed three days, and was suffering from the ordinary symptoms of dysentery, with fever, pain and discharges of blood, but combined with extreme debility and prostration, so as to cause great apprehensions for the result. The severer symptoms yielded readily to the treatment, and his medical attendants flattered themselves with the hope that he might still be spared, but these hopes proved delusive; the disease, though not severe, had exhausted the little strength which he possessed, and there seemed no power of restoration. Every effort was made to sustain the system, (which was all that could be done) but these efforts were all vain, and he expired without a struggle or a groan, on the morning of the 8th of September, 1849. The Doctor himself, from the first, said he should not recover, spoke calmly but freely about his death, gave directions about his affairs, and as to his burial, requesting to be laid beside his beloved son, and that the bodies of both should subsequently be removed to the new cemetery, where a spot has been selected for their interment.

Dr. Brigham was a philanthropist, a lover of his brother man, in the strictest sense of the term; he no doubt was ambitious of fame and distinction, but he was still more ambitious of being useful, and often expressed the idea that he saw no object in living, after a man had ceased to be useful. Fortunately for the community, the usefulness of which he was most ambitious, will not perish with him. As the first Superintendent and organizer of the New York State Lunatic Asylum, he has erected a monument as durable as the blocks of stone of which it was built. His teachings, too, live in his writings. In addition to his annual reports, in which the whole subject of insanity is discussed, and the editorial articles in the "Journal of Insanity," he has at different times published works of a more permanent character. In 1832 he published a small volume on the epidemic or Asiatic cholera; also a work on mental cultivation and excitement. In 1836, a volume on the influence of religion upon the health and physical welfare of mankind. In 1840, a volume on the brain, embracing its anatomy, physiology and pathology. His last publication was an appropriate crowning of his labor of benevolence; it is a small duodecimo volume, entitled "The Asylum Souvenir," and is dedicated to those who have been under the care of the author and compiler. It consists of a collection of aphorisms and maxims, to aid in the restoration and preservation of health, and we have no doubt it will be cherished with a double care, as it may now be considered the parting legacy of their friend and benefactor. The following extracts will exhibit the general tenor of the work:—

"THE ASYLUM SOUVENIR.

To all those who are or have been in my charge as patients, this little book is affectionately dedicated by their friend, AMARIAH BRIGHAM.

---

Peace be around thee wherever thou rovest;
May life be for thee one summer's day,
And all that thou wishest, and all that thou lovest,
Come smiling around thy sunny way.
If sorrow e'er this calm should break,
May even thy tears pass off so lightly,
Like spring showers, they'll only make
The smiles that follow, shine more brightly."

Were we asked what were the leading traits in the character of our departed friend, we should answer, that the first and strongest impulse, was one of kindness and benevolence, but this was combined with a high sense of justice, and he would not indulge the former at the expense of the latter. In addition, he possessed a strong feeling of self-reliance, a quickness of perception which enabled him to seize readily the views of others, and use them for his own purpose; but above and before all, an iron will and determination, which brooked no opposition, consequently in whatever situation he was placed, he must be absolute, or he was unhappy. It is seldom we find this strong determination of purpose connected with a feeble constitution, but whenever it exists, the individual may be marked for a premature grave: the strongest constitution can scarcely long maintain itself under the thousand irritations and annoyances to which such a will is subject.

The following extracts from the reports of Dr. B., while Superintendent of the Asylum at Utica, are a specimen of the tone of kindness which pervades all his writings:—

"That education which consults the good of the whole man, that tends to develope and strengthen, in just proportion, the moral, intellectual and physical powers, is conducive to health of body and mind. But in all countries the intellect or some of the intellectual faculties are cultivated to the neglect of the moral qualities, while in others, the feelings, appetites and propensities, are too greatly indulged and cultivated, to the neglect of just intellectual improvement. Hence arise unbalanced minds which are prone to become disordered. They feel too intensely, and are too ardently devoted to the accomplishment of certain purposes, to bear disappointment without injury. They have not been taught *self-denial*, without which, all education is defective.—3d ann. rep. pp. 54, 55.

"Allusion has been made to a predisposition to insanity being given by premature cultivation of the mental faculties. This appears to be a fruitful source of weak, ill-regulated, and not unfrequently, disordered minds. The mental powers being unduly and irregularly tasked in early life, never after obtain their natural vigor and harmonious action. The dominion of reason should extend over all the feelings and impulses, the good as well as the bad, for insanity is perhaps most frequently produced by the excitement of some of the best impulses of our nature."—1st report, pp. 34, 35.

Dr. Brigham, as we have said, was ambitious, but his was a noble ambition. He was ambitious of being useful to mankind, and of leaving a monument by which he should be remembered in after ages, and be ranked among the benefactors of our race; and most nobly has he succeeded. Few men were less covetous of personal popularity, or more regardless of the opinions of those about him, so long as he was sustained by the approbation of his own conscience. The following extract from Bryant, which he himself selected for "The Asylum Souvenir" but a short time before his death, beautifully expresses the purpose of his life, and the manner of his death:—

So live, that when thy summons comes to join
The innumerable caravan, that moves
To that mysterious realm, where each shall take
His chamber in the silent halls of death,
Thou go not like the quarry-slave at night,
Scourged to his dungeon, but sustain'd and sooth'd
By an unfaltering trust, approach thy grave,
Like one who draws the drapery of his couch
About him, and lies down to pleasant dreams.

